# Underscoring the challenges in the training of kidney transplant surgeons

**DOI:** 10.1590/2175-8239-JBN-2024-E011en

**Published:** 2024-11-11

**Authors:** Renato Demarchi Foresto, Lúcio Requião-Moura

**Affiliations:** 1 Universidade Federal de São Paulo, São Paulo, Brazil.; 2 Fundação Oswaldo Ramos, Hospital do Rim, São Paulo, Brazil.

In 2023, 14,397 patients were listed for a kidney transplant in Brazil, but only 6,047 transplants were performed^1^. While not all dialysis patients are clinically eligible for transplantation, the demand for organs is still far from being met. Traditionally, discussions about increasing the number of transplants in Brazil and globally focus on strategies to expand the donor pool, such as using organs from deceased donors with high kidney donor profile index (KDPI), acute kidney injury, or prolonged cold ischemia time, and defining strategies for transplantation with serological matching between donor and recipient in patients living with HCV or HIV^2,3^. However, more discussions about the scope and quality of training of new professionals capable of meeting this demand are needed.

According to data from the Brazilian Transplant Registry, there are 146 active kidney transplant teams in Brazil, with an average of 41 transplants per team, and 18% of these teams routinely perform at least one transplant per week^1^. Additionally, there is a significant geographical disparity in the distribution of these teams among states, with a concentration in the South and Southeast regions^1^. On the other hand, there are 5,820 urologists with active specialty registration in the country and 215 residency spots available annually, leading to a 64% increase in the number of specialists over the last decade despite a vacancy rate of around 30% in medical residency slots for this specialty^4^.

However, for a urologist to undergo formal training in kidney transplantation, they must complete three years of general surgery residency, followed by three years of urology and an additional year in kidney transplantation. This lengthy training, coupled with the apparent limited financial attractiveness of the transplant field compared with other subspecialties within urology, discourages the engagement of young professionals. As a result, the available residency spots in kidney transplantation for urology often remain unfilled, and in some centers, there have been no candidates in recent years.

The study “An analysis of fellowship training of kidney transplant surgeons in a Brazilian state” by Ferreira et al.^5^ highlighted the challenges faced, reflecting the local reality and the urgent need for reforms in the training and working conditions of these professionals across the country. The study involved 17 active kidney transplant centers and focused on the training and practices of transplant surgeons in a Brazilian state. Although it was conducted in centers from only one Brazilian state, the epidemiology may reflect the situation of the specialty nationwide. The predominance of men (95%) and the average age of 46.3 years reflect the specialty’s characteristics with a significant focus on men’s health. They were consistent with the sex and age distributions found nationally for urology^4^. A large portion of the centers was in the state capital (47%), with significant participation from educational institutions (88%) that provide care through the public health system, reinforcing the importance of the public system in funding transplants^5^.

Among the activities of transplant surgeons, organ procurement surgery is particularly unappealing for various reasons, including the urgent nature, the often long distances between hospitals, and the low remuneration. A solution would be the inclusion and proper training of general surgeons in organ procurement activities. This could quickly optimize transplant teams and create a new niche for these young surgeons, reducing the current 7-year training path to three years of residency. Similarly, this movement should be discussed within medical societies to create a career path for transplant surgeons similar to thoracic or abdominal transplant surgeons, as seen in many other countries.

Furthermore, another concerning finding from the study by Ferreira et al.^5^ is that only 25.6% of surgeons reported completing some form of residency or specialization in transplantation. Despite the specialty being among the least sought after by newly graduated doctors due to the intense commitment required by the training and routine of a transplant surgeon and its unfavorable market position compared with other urology subspecialties, 95% of transplant surgeons in the US, where there are 66 transplant training programs^6^, have specialized training.

Although Brazilian surgeons mention the lack of specific transplant residency programs as a justification for not seeking training, the vacancies in transplant residency spots and the belief of some surgeons that training is unnecessary contradict this first statement. Once again, one of the barriers of the Brazilian transplant program is the geographic disparity in the distribution of human resources for the donation and transplant process. Increasing the availability of places in transplant residency could help recruit young professionals into the specialty. Therefore, the regulations of residency programs must also be reviewed and minimum training requirements must be defined to ensure the quality of education. This step seems to be essential to formally recognize kidney transplant surgery as a specialty and improve training and working conditions to create well-trained teams.

The study on the training and performance of kidney transplant surgeons in Minas Gerais offers a critical view of this current and relevant issue for our society^5^. The findings underline the need for significant reforms in the training structure and working conditions with the aim of improving the quality of transplants and increasing the availability of specialized surgeons. The medical community and those responsible for health policy in Brazil must collaborate to overcome these challenges and ensure that patients receive the best possible care.
