# Supplementary material for: Underscoring the challenges in the training of kidney transplant surgeons
Source: J Bras Nefrol. 2024 Nov 11;46(4):e2024E011. doi: 10.1590/2175-8239-JBN-2024-E011en (PMC11588315; doi:10.1590/2175-8239-JBN-2024-E011en)
Supplement: Translation

# Translation: Destacando os desafios na formação de cirurgiões de transplante renal

**DOI:** 10.1590/2175-8239-JBN-2024-E011pt

**Authors:** Renato Demarchi Foresto, Lúcio Requião-Moura

**Affiliations:** 1 Universidade Federal de São Paulo, São Paulo, Brasil.; 2 Fundação Oswaldo Ramos, Hospital do Rim, São Paulo, Brasil.

Em 2023, 14.397 pacientes foram listados para transplante renal no Brasil, mas somente 6.047 transplantes foram realizados^1^. Embora nem todos os pacientes em diálise sejam clinicamente elegíveis para transplante, a demanda por órgãos ainda está longe de ser atendida. Tradicionalmente, as discussões sobre o aumento do número de transplantes no Brasil e no mundo concentram-se em estratégias para ampliar o *pool* de doadores, como a utilização de órgãos de doadores falecidos com alto índice de perfil de doadores de rim (KDPI, por sua sigla em inglês), injúria renal aguda ou tempo de isquemia fria prolongado, além da definição de estratégias para transplante com compatibilidade sorológica entre doador e receptor em pacientes portadores de HCV ou HIV^2,3^. No entanto, é necessário promover mais discussões sobre o escopo e a qualidade da formação de novos profissionais capazes de atender a essa demanda.

Segundo dados do Registro Brasileiro de Transplantes, existem 146 equipes de transplante renal ativas no Brasil, com uma média de 41 transplantes por equipe, sendo que 18% dessas equipes realizam rotineiramente pelo menos um transplante por semana^1^. Além disso, há uma disparidade geográfica significativa na distribuição dessas equipes entre os estados, com uma concentração nas regiões Sul e Sudeste^1^. Por outro lado, existem 5.820 urologistas com registro de especialidade ativo no país e 215 vagas de residência disponíveis anualmente, resultando em um aumento de 64% no número de especialistas na última década, apesar de uma taxa de vacância de cerca de 30% nas vagas de residência médica para essa especialidade^4^.

No entanto, para que um urologista receba treinamento formal em transplante renal, ele deve concluir três anos de residência em cirurgia geral, seguidos de três anos em urologia e mais um ano em transplante renal. Esse longo período de formação, aliado à aparente atratividade financeira limitada da área de transplante em comparação com outras subespecialidades dentro da urologia, desestimula o engajamento de jovens profissionais. Como resultado, as vagas disponíveis de residência em transplante renal para urologia frequentemente não são preenchidas e, em alguns centros, não houve candidatos nos últimos anos.

O estudo “Uma análise do treinamento *fellowship* de cirurgiões de transplante renal em um estado brasileiro”, de Ferreira et al.^5^ destacou os desafios enfrentados, refletindo a realidade local e a necessidade urgente de reformas na formação e nas condições de trabalho desses profissionais em todo o país. O estudo envolveu 17 centros ativos de transplante renal e concentrou-se no treinamento e nas práticas dos cirurgiões de transplante em um estado brasileiro. Embora tenha sido realizado em centros de apenas um estado do Brasil, a epidemiologia pode refletir a situação da especialidade em todo o país. A predominância de homens (95%) e a média de idade de 46,3 anos refletem as características da especialidade, com foco considerável na saúde masculina. Esses dados foram consistentes com as distribuições de sexo e idade encontradas nacionalmente para a urologia^4^. Grande parte dos centros estava localizada na capital do estado (47%), com participação significativa de instituições de ensino (88%) que oferecem atendimento por meio do sistema público de saúde, reforçando a importância desse sistema no financiamento de transplantes^5^.

Entre as atividades dos cirurgiões de transplante, a cirurgia de captação de órgãos é particularmente pouco atrativa por vários motivos, incluindo sua natureza urgente, as frequentemente longas distâncias entre os hospitais e a baixa remuneração. Uma solução seria a inclusão e o treinamento adequado de cirurgiões gerais nas atividades de captação de órgãos. Isso pode otimizar rapidamente as equipes de transplante e criar um novo nicho para esses jovens cirurgiões, reduzindo o atual período de 7 anos de formação para três anos de residência. Da mesma forma, esse movimento deve ser debatido nas sociedades médicas a fim de estabelecer uma trajetória profissional para os cirurgiões de transplante semelhante àquela dos cirurgiões de transplante torácico ou abdominal, como observado em muitos outros países.

Além disso, outro achado preocupante do estudo de Ferreira et al.^5^ é que somente 25,6% dos cirurgiões relataram ter concluído alguma forma de residência ou especialização em transplante. Apesar de a especialidade estar entre as menos procuradas por médicos recém-formados, devido ao intenso comprometimento exigido pela formação e rotina de um cirurgião de transplante, além de sua posição desfavorável no mercado em comparação com outras subespecialidades da urologia, 95% dos cirurgiões de transplante nos EUA, onde existem 66 programas de treinamento em transplante^6^, possuem formação especializada.

Embora os cirurgiões brasileiros mencionem a falta de programas específicos de residência em transplante como justificativa para não buscarem treinamento, as vagas em programas de residência em transplante e a crença de alguns cirurgiões de que a formação é desnecessária contradizem essa primeira afirmação. Mais uma vez, uma das barreiras do programa brasileiro de transplantes é a disparidade geográfica na distribuição de recursos humanos para o processo de doação e transplante. Aumentar a disponibilidade de vagas na residência em transplante pode ajudar a recrutar jovens profissionais para a especialidade. Portanto, as regulamentações dos programas de residência também devem ser revisadas e os requisitos mínimos de formação devem ser definidos para garantir a qualidade do ensino. Essa etapa parece ser essencial para reconhecer formalmente a cirurgia de transplante renal como uma especialidade e melhorar o treinamento e as condições de trabalho com o objetivo de criar equipes bem treinadas.

O estudo sobre a formação e o desempenho de cirurgiões de transplante renal em Minas Gerais oferece uma visão crítica dessa questão atual e relevante para a nossa sociedade^5^. Os achados ressaltam a necessidade de reformas significativas na estrutura de treinamento e nas condições de trabalho, com o objetivo de melhorar a qualidade dos transplantes e aumentar a disponibilidade de cirurgiões especializados. A comunidade médica e os responsáveis pela política de saúde no Brasil devem colaborar para superar esses desafios e garantir que os pacientes recebam o melhor atendimento possível.
